# Advice for translational neuroscience: move deliberately and build things

**DOI:** 10.1186/s42234-025-00165-w

**Published:** 2025-02-03

**Authors:** Seth A. Hays, Robert L. Rennaker, Michael P. Kilgard

**Affiliations:** 1 Texas Biomedical Device Center, University of Texas at Dallas, Richardson, TX 75080 USA; 2 Department of Bioengineering, Erik Jonsson School of Engineering and Computer Science, University of Texas at Dallas, Richardson, TX 75080 USA; 3 Department of Neuroscience, School of Behavioral and Brain Sciences, University of Texas at Dallas, Richardson, TX 75080 USA

Real innovations in medicine and science are historic and singular; the stories behind each occurrence are precious but often go untold. At *Bioelectronic Medicine* we have established the Anthony Cerami Award in Translational Medicine to document and preserve these histories. The monographs recount the seminal events as told in the voice of the original investigators who provided the crucial early insight. These essays capture the essence of discovery, chronicling the birth of ideas that created new fields of research; and launched trajectories that persisted and ultimately influenced how disease is prevented, diagnosed, and treated. Here, the Cerami Award Monograph is by Seth A. Hays, Robert L. Rennaker, and Michael P. Kilgard – leaders in using vagus nerve stimulation in the treatment of neurological conditions, including poor hand function after a stroke. This is the story of their scientific journey.

In light of the fact that this is the first Anthony Cerami Award for Translational Medicine shared by investigators for collaborative work, the following monograph reflects this unique position (Fig. 1). In this text, we focus less on our individual pathways and more on the nature of and lessons from working collectively towards a major research goal. We provide a historical perspective on our collaborative research enterprise, and we discuss the guiding principles, both those that succeeded and those that did not. Though certainly not intended as an all-encompassing template for team science, we hope that this narrative can provide some context to guide those interested in pursuing a collaborative, unified approach.

**Fig. 1:**
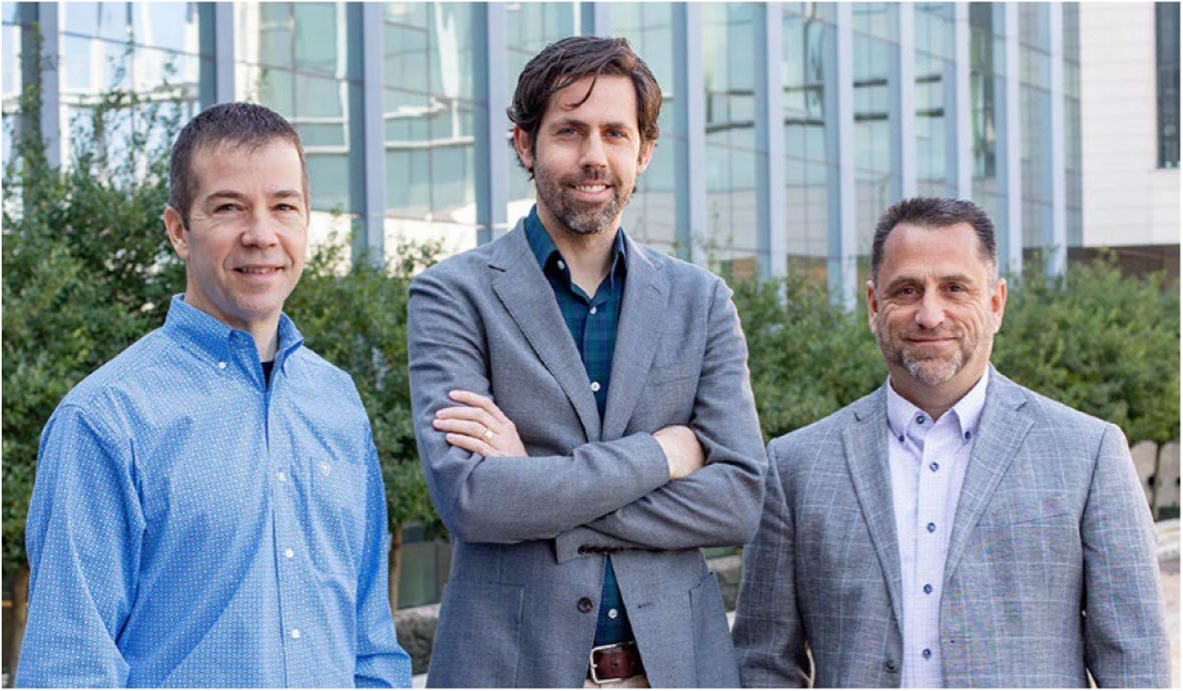
Cerami Award Recipients. From left: Michael Kilgard, Seth Hays, and Rob Rennaker

## Origins of targeted plasticity

The ability to direct specific changes in communication between neurons, a process referred to as plasticity, and thereby influence the function of neural circuits and ultimately behavior, is a long-standing goal in neuroscience. In principle, this approach could be used to normalize neural activity in circuits affected by injury or disease, with the ultimate goal of restoring health (Hays et al. 2013).

A great many strategies have been developed and tested to direct robust, specific plasticity, many of which are premised on increasing the action of neuromodulators to facilitate the changes between neurons activated by training (Frémaux and Gerstner 2015). The origins of our use of such a targeted plasticity approach derive from Mike’s early work combining presentation of sounds, which produce neural activity in networks that process auditory information, with electrical stimulation of the nucleus basalis, a forebrain structure that governs release of acetylcholine, a neuromodulator.

While working as a graduate student under the supervision of Dr. Michael Merzenich, Mike’s initial experiments in rats demonstrated that combining short bursts of nucleus basalis stimulation (NBS) could elicit long-lasting plasticity in auditory networks that was specific to the nature of the paired sounds (Kilgard et al. 1998; Kilgard et al. 1998). Pairing low sounds with NBS increased the number of neurons in the brain that respond to low sounds. Pairing a sequence of sounds with NBS increased the number of neurons that respond to that sequence of sounds (Kilgard et al. 2002). These experiments proved out the concept of using electrical stimulation of neuromodulatory structures to produce specific forms of plasticity, the fundamental concept of targeted plasticity.

Upon completing his PhD, Mike started his own laboratory at the University of Texas at Dallas that expanded on the themes of targeted plasticity. Early experiments provided further confidence that this strategy for manipulating plasticity could produce meaningful changes in behavior (Reed et al. 2011), but one aspect of nucleus basalis stimulation loomed that would limit the goal of using this approach in humans. The nucleus basalis is located at the base of the forebrain, thus activation requires implantation of a deep brain stimulating electrode. While not strictly disqualifying, Mike recognized the potentially restrictive impact and began seeking an alternative approach.

An ideal solution would provide similar engagement of neuromodulatory networks, retain the precise timing afforded by electrical stimulation, and reduce the invasiveness needed for reliable stimulation. These features led to investigation of vagus nerve stimulation (VNS) to replace nucleus basalis stimulation. A series of compelling prior studies showed that stimulation of the vagus nerve, either electrically or chemically, could facilitate memory, an effect clearly ascribed to enhancement of plasticity (Jensen 1996; Williams 1991; Williams et al. 1993; Clark et al. 1999; Clark et al. 1995; Clark et al. 1998; Flood et al. 1987; Zuo et al. 2007). Moreover, a small body of literature suggested that these effects were mediated by VNS actions on neuromodulatory networks (Krahl et al. 1998; Roosevelt et al. 2006; Nichols et al. 2011). Perhaps most importantly, however, VNS received FDA approval for the treatment of epilepsy in1997and had been widely used and well-tolerated in patients (Ben-Menachem 2002).

This conceptual basis led to direct evaluation of VNS as a means to enhance plasticity. In a landmark study for the development of VNS-based targeted plasticity, a talented postdoctoral scholar in Mike’s lab showed that VNS paired with presentations of tones could direct robust, specific plasticity in auditory cortex in rats (Engineer et al. 2011). These findings revealed that short bursts of VNS could replicate the magnitude and nature brain changes observed with NBS while obviating the need for implanting a deep brain electrode. Moreover, VNS pairing produced improvements in measures of tinnitus, highlighting the potential of using targeted plasticity to impose a meaningful effect on recovery.

In parallel, Rob was following a much different path to science. After his distinguished service in the U.S. Marine Corps from 1988 to 1993 in Operations Sharp Edge, Desert Shield/Storm, and Provide Comfort, Rob pursued his passion for engineering and healthcare, obtaining a PhD in Biomedical Engineering from Arizona State University in 2002 in Dr. Daryl Kipke’s lab. Rob’s work focused on understanding neural processing and plasticity in auditory cortex of awake behaving rodents, which led him to a postdoctoral stint with Mike prior to accepting a faculty position at The University of Oklahoma. From 2002 to 2009, Rob and Mike collaborated on several projects and upon demonstration of the potential of VNS-based targeted plasticity therapy, Rob came to UT Dallas to work with Mike and make a unified effort to bring this technology to clinical use.

Our initial studies using paired VNS to promote plasticity were largely focused on the auditory system, a choice predominantly driven by Mike’s background (Engineer et al. 2011; Shetake et al. 2011; Engineer et al. 2015). However, these findings raised the prospect that a congruent strategy of combining VNS with motor training would similarly drive specific plasticity in motor networks. The ability to direct plasticity in motor networks holds immense clinical potential, as this has long been recognized as a substrate for recovery after neurological injuries, such as stroke (Murphy et al. 2009).

To test this principle, it was first necessary to develop a motor task that would permit reliable, preferably automated, triggering of VNS during movement. The existing motor behavioral tasks employed in rats had been effectively used in a number of labs, but they were labor-intensive and stood to benefit from modernization. In an early example of the importance of complementarity discussed below, Rob brought to bear his engineering training to develop a set of motor tasks that could support paired delivery of VNS (Hays et al. 2013; Hays et al. 2013; Meyers et al. 2016). Using some of these tools, a key early study demonstrated that combining VNS with motor training promoted specific plasticity in the networks activated by the training (Porter et al. 2011).

The desire to test VNS paired with motor training and the development of these motor tasks coincided with Seth’s arrival at UT Dallas. Seth had two elements that made the union a good fit: prior experience with motor research and a general dissatisfaction with the hyper-reductionist focus of most biomedical research that supplanted translation. Collectively, we set about testing whether VNS combined with motor training could promote plasticity and improve recovery in a rat model of stroke. The first study yielded intriguing positive results, which ramped up the development of paired VNS therapy for stroke in earnest (Khodaparast et al. 2013).

## Inception of the Texas Biomedical Device Center

Recognizing the potential of targeted plasticity, leadership at UT Dallas, led by Dr. Hobson Wildenthal, and an anonymous donor from Texas Instruments provided critical investment in this approach by endowing the Texas Biomedical Device Center (TxBDC) in 2013. The infrastructure provided by TxBDC allowed us to truly streamline our work and align our efforts to focus squarely on bringing this approach to its hopeful conclusion: impact on patients.

The main initial thrust of TxBDC was to develop and translate VNS therapy for stroke. Over a series of preclinical experiments, we built on the initial success to demonstrate that VNS combined with rehabilitation improves recovery in common complicating clinical situations, like advanced age or a delay before therapy initiation (Khodaparast et al. 2014; Khodaparast et al. 2016; Hays et al. 2016; Hays et al. 2014; Hays et al. 2014; Morrison et al. 2022). We also found that VNS-dependent recovery generalized to other similar movements, a key consideration in designing rehabilitative regimens (Meyers et al. 2018). These efforts also revealed neural changes, namely the increase in connectivity brain circuits that control the rehabilitated limb (Meyers et al. 2018). Lastly, to support the transition to human use, we sought to optimize the stimulation paradigms, a process that would be a clear requirement for pharmacological therapies, but is often overlooked for bioelectronic therapies despite evidence of its importance. Through the course of optimization, we identified a select set of parameters that were effective and many that were not (Pruitt et al. 2021; Morrison et al. 2019; Morrison et al. 2020; Morrison et al. 2021). As described further below, we believe that this mapping of the range of effective conditions to be a critical step in productive translational research.

This preclinical research set the stage for clinical success. As VNS progressed toward clinical use, Seth was recognized by the American Heart Association with the Robert G. Siekert New Investigator for Stroke award in 2015. The start-up company MicroTransponder licensed the VNS therapy technology for stroke from UT Dallas and brought the technology to clinical investigation. Through a series of progressing clinical studies culminating in a large pivotal study, they and a large, talented group of clinician researchers demonstrated that VNS therapy is safe and effective (Dawson et al. 2016; Kimberley et al. 2018; Dawson et al. 2021). This work led to VNS therapy becoming the first ever FDA-approved treatment to improve recovery in individuals with chronic stroke in 2021 (FDA Approves First-of-Its-Kind Stroke Rehabilitation System | FDA n.d.). Beyond the direct clinical importance, this both points to the utility of the preclinical studies that validated the concept for treating neurological disorders with targeted plasticity.

## Expansion of targeted plasticity therapy

**Fig. 2:**
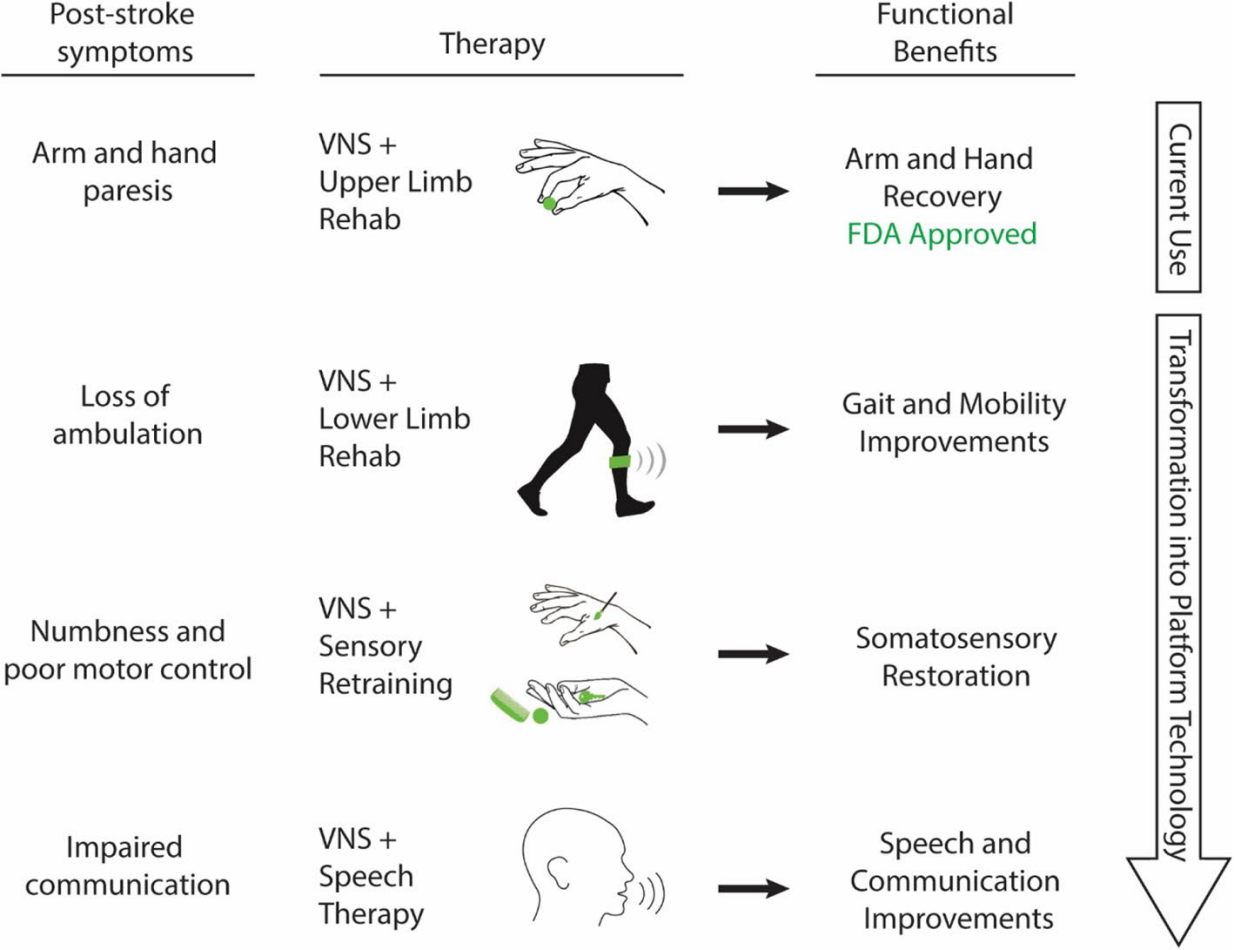
VNS Therapy as a Platform Technology

In principle, VNS therapy represented a platform technology to flexibly address a range of neurological conditions if combined with the appropriate form of rehabilitation. This is exemplified in our early experiments showing that VNS paired with sounds in the auditory system, while using VNS paired with movement to engender changes in motor networks signaled the potential of this approach as a platform technology. Consequently, it is conceivable that pairing VNS with other known rehabilitation strategies could target other common post-stroke disabilities (Fig. 2). Beyond this, if combining VNS with training drives training-specific plasticity in relevant networks, could we apply this approach to other neurological conditions with other forms of therapy (Hays et al. 2013; Engineer et al. 2019)?

Our ability to pursue this question was facilitated by the fortuitous launch of the Defense Advanced Research Projects Agency (DARPA) ElectRx program, which sought to use bioelectronic medicine to target treatment of a disease of relevance to the Department of Defense. We used this opportunity to stretch the potential of paired VNS. To this point, our use of VNS had focused predominantly on delivering stimulation concurrent with discrete, quantifiable events, like a movement or a sound, to directly target synaptic changes related to these events. However, we had speculated that a similar principle may also facilitate plasticity when combined with complex, continuous events, but we had yet to test it. Specifically, we sought to explore the ability of VNS to influence fear extinction learning, a common laboratory technique thought to be relevant to the treatment of post-traumatic stress disorder (PTSD).

Fear extinction requires the formation of new memories to overwrite the memories associated with cues that evoke fear (Fanselow and Fanselow 2013; Noble et al. 2019). Early work showed electrical stimulation of the vagus nerve could influence extinction learning (Peña et al. 2012; PeñA 2014). With the support of the ElectRx project, we leveraged the collective strength of our team to evaluate, develop, and translate this potential in the context of the treatment of PTSD. The project encompassed technology engineering and development, preclinical optimization, and human clinical trials, which allowed us to both use and grow our complementary expertise in these areas (Souza et al. 2020; Souza et al. 2021; Noble et al. 2019; Souza et al. 2021; Souza et al. 2022; Souza et al. 2019; Noble et al. 2017; Mathew et al. 2020; Noble et al. 2019). The culmination of this effort was the development and translation of a fundamentally new therapy for treatment-resistant PTSD, which is now progressing towards a pivotal trial.

During the conduct of the ElectRx project, a second DARPA program, entitled Targeted Neuroplasticity Training (TNT), was released. Coincidentally, this program fit squarely within our expertise, and by distributing the effort across the team, we agreed that we had the bandwidth and infrastructure to support participation in this program. Our project was selected for funding, and the TNT program provided two critical elements to the development of VNS therapy. First, the TNT program allowed us to expand the use of VNS paired with motor training to improve recovery of motor function in the context of spinal cord injury and supported a clinical trial of this approach in partnership with Wings for Life, which built the foundation for an upcoming pivotal trial of this approach (Sachdeva et al. 2020; Darrow et al. 2020; Ganzer et al. 2018). Second, and less directly clinically applicable but perhaps more irreplaceable given the unusual nature and “freedom to fail” ethos of DARPA, was support for parameterization of VNS therapy. Specifically, this project motivated and supported the wide-ranging evaluation of VNS therapy across dozens of stimulation parameters, electrode designs, training paradigms, and therapy schedules (Morrison et al. 2019; Morrison et al. 2020; Morrison et al. 2021; Buell et al. 2018; Loerwald et al. 2018; Buell et al. 2019; Borland et al. 2016; Loerwald et al. 2018; Hulsey et al. 2017; Darrow et al. 2021; Ruiz et al. 2023; Ruiz et al. 2023; Malley et al. 2024; Bucksot et al. 2020; Bucksot et al. 2019). These studies were essential to the translation of VNS therapy and, given their expressly incremental nature, would have been absolute non-starters at conventional funding agencies.

Collectively, these DARPA projects were only achievable, both from the perspective of obtaining funding but in terms of making substantial progress, because we had chosen to work as a team. The progress over the course of this decade after the founding of TxBDC really provided the necessary momentum to bring these treatments to patients.

## From bench to bedside

A major shift in the focus of TxBDC came to fruition in early 2020. We had always been keenly focused on performing science that is directly useful for translation, but our work had largely been restricted to engineering and preclinical work in the lab. The support from DARPA allowed us to expand into human trials of VNS therapy.

**Fig. 3:**
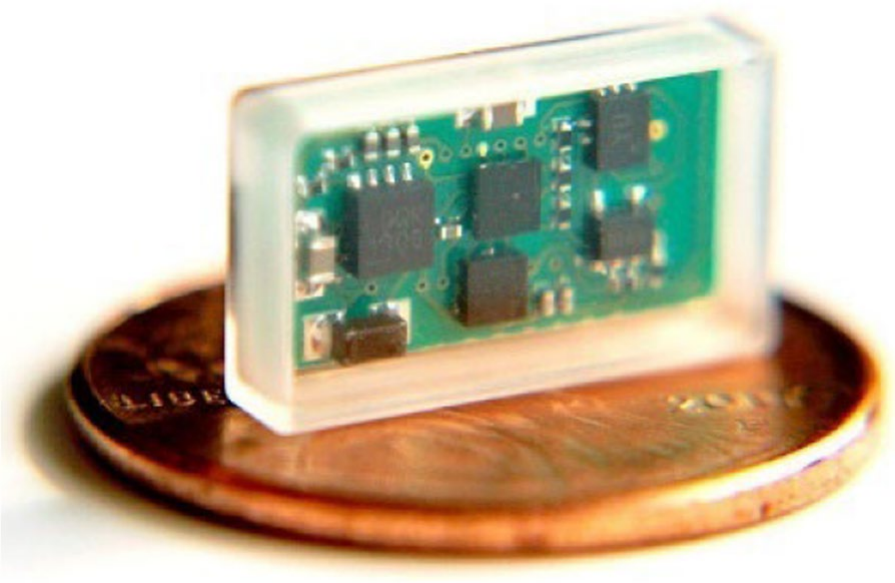
The TxBDC miniaturized VNS system

A key decision, which was largely spearheaded by Rob and involved a considerable amount of risk, was critical to clinical translation and exemplifies the importance of trusting your collaborators. Conventional VNS devices had been around for over two decades when we began our initial clinical trials, and a triggerable version which would allow delivery of paired VNS therapy had been developed by MicroTransponder. Rob recognized that access to a stimulator would obviate any issues with being beholden to a supplier, allowing significant freedom and control over the use and data associated with the device. With support from the W. W. Caruth Jr. Foundation, we elected to invest in the development of our own VNS device, which imposed significant costs and burden of effort (Sivaji et al. 2019). Ultimately, the miniaturized VNS system borne from these efforts allowed us to capitalize on the advances in technology since the original VNS systems and, perhaps more importantly, conferred unfettered access to this system to broadly develop VNS therapy without external restrictions (Fig.3).

With the next-generation VNS system in hand, we undertook three first-in-human clinical trials effectively simultaneously (which we would not necessarily recommend). To evaluate the utility of the miniaturized VNS device, we replicated the original clinical study of VNS therapy in chronic stroke patients (ClinicalTrials.gov Identified: NCT04534556). Additionally, we expanded to test the safety and feasibility of VNS therapy to improve arm and hand recovery in individuals with incomplete spinal cord injury in a randomized, double-blinded study (NCT04288245). Lastly, we performed an open label study to evaluate a congruent approach of pairing VNS with prolonged exposure therapy in individuals with chronic, treatment resistant PTSD (NCT04064762). These studies are now complete and results are presently forthcoming, but across fifty implanted individuals, we observed that VNS therapy is a platform therapy that is safe, feasible to deliver, and yields clinically-meaningful improvements. Our current efforts are directed at maximizing efficacy in these patients and bring VNS therapy forward to pivotal trials for these conditions, which forms the basis for FDA approval and eventual clinical implementation.

## Reflections on team science

We have taken a serious, deliberate approach to team science that has been fruitful, and below we will expound on a few elements that have been integral. That said, we certainly wish to disclaim that we have arrived at some special knowledge and that these points should not to be interpreted as the guidelines for “doing it right”, whatever that would even mean. Additionally, some of these will sound like platitudes, which reflects them just generally being good ideas that survived the tests of time and that we subsumed. Ultimately, we simply seek to provide our collective experiences and any received wisdom regarding what has and hasn’t worked for us as it relates to team science.

### Selecting projects

Perhaps the most fundamental, though rarely examined, aspect of a research program is the selection of projects. This occurs across a number of levels, from something as broad and abstract as the overall mission of a research program (for example, our focus on translational research for treating neurological and psychiatric disease), to the concrete and minute (such as whether to include four or six conditions in a dosing experiment). At the risk of being hyperbolic, these choices can, in some instances, have tremendous consequences on a research program writ large, and all the individuals involved in the process. Often, these choices are driven by whims and vagaries; perhaps we are taking a measured approach to maximize the impact factor of an eventual publication, but often scientists seek to select projects that are simply the coolest, or maybe driven by some recency bias in a talk we just heard.

To mitigate this randomness, we have codified a quantitative approach to project selection that attempts to balance three factors crucial for the utility of any project. This process is iterative and can be time consuming, but is nevertheless a useful tool for organizing thoughts and forcing the issue on deeply considering projects. The first step is to develop a list of fairly well articulated potential projects. The length of the list will vary based on the level of familiarity of the listmaker, for example, a new graduate student might arrive at a list of ten, whereas a principal investigator may curate a list of fifty. Longer is better in this case, as it directly relates to the quality of the eventual ranking. If you select the best from a list of 10, your project will be in the top 10%; if you do the same from a list of 100, it will be in the top 1%. Each project should have a sufficient level of detail to determine the number of conditions, groups, and projected sample sizes.

Once you have established a list of projects, each project is given a numerical ranking between zero and one in three categories, defined simply as ‘easy’,’ likely’, and ‘care’. Easy is, as the name suggests, a measure of how easy a project will be to complete. This category should account for the total number of samples and conditions, the complexity and time of the experimental procedures, the amount of data already collected (if any, for example, in a pilot study) and can include cost as a factor, as relevant. Projects that are easier to accomplish are given higher scores, and one should strive for a linear relationship in scoring. By means of an example, if an experiment has twice as many groups, all else being equal, it should have half the score as the smaller project. The second category, Likely, describes how probable a project is to produce a clear and useful outcome, regardless of whether the null hypothesis is rejected or confirmed. This does not necessarily mean the likelihood of arriving at the most interesting outcome (i.e., “get it to work”) to score well; clear negative results are equivalently, if not more, valuable to increase understanding and drive new research avenues. Projects that are more likely to yield a useful and unambiguous outcome are given higher scores. Care, the third category, is more subjective. This score can be driven by the current utility of the project; we often score this category based on how probable the outcome is to directly influence patient care, the impact of the eventual publication, and whether it will produce useful preliminary data for a grant. Projects that are poised to make a higher impact are given higher scores. Here, too, we strive to be linear, which means that projects with twice the potential impact should have twice the score. Once scoring is complete, it is useful to rank each category and compare individual projects to one another to ensure consistency and linear scaling.

Once the list is assembled and scored, the next step is to generate an overall ranking. The category scores for each project are multiplied to produce a single value, and the list is re-ranked based on this calculated score. The top project represents the optimal choice. This approach seems bizarre and time consuming in the beginning, but we believe brings significant clarity to the process. This is not intended to be a static process. Keeping a curated list and updating it when relevant can be useful. Though it may seem that continuous adjustment would introduce instability, this tends to not be the case. For example, if a project is halfway complete and initial analysis indicates that it will produce meaningful results, its ‘easy’ and ‘likely’ scores should be correspondingly increased. However, it does allow emerging better projects to displace dead wood. If evolving evidence from recent studies provides new context, or a project is proving more challenging than expected, or the preliminary results foreshadow an unlikelihood to produce actionable evidence, then this project, even if ongoing, *should* be paused in lieu of a better choice. Though this can seem antithetical to progress (and can be frustrating for students if not justified appropriately), it is in everyone’s best interest to avoid the Concorde Fallacy and do the best possible science.

This approach has forced us to stay intellectually honest and mitigates the selection bias in choosing projects. Moreover, a clear codification of the approach is useful, in that ensures the team is on the same page and focused on similar goals. The outcome is not to homogenize thinking (we can certainly confirm that this is not the case), but rather to stimulate discussion and produce consensus on a choice as critical as project selection.

Separately formalizing the amount of effort to complete each project, the likelihood of success, and the impact of success offers the additional benefit that it enables active participation of all members of the team. For example, graduate students and technicians may have a better estimate of how long certain experiments will take to complete than more senior investigators might. Linearly ordering each possible project on each measure before examining their multiple helps ensure decisions are based on realistic estimates that are widely agreed up without the contamination that occurs from seeing that a given project is the frontrunner. The highest value is almost always a surprise to the team doing the analysis, which is an indication that confirmation bias is not the primary driver of project selection decisions.

### Defining the conditions that don’t work

A crucial step in development and translation is to define the conditions over which the therapy does not work. This lesson is driven by our focus on translational research and has been proposed, in some form, from various other sources (for example, see (Lapchak et al. 2013)). These experiments, which for simplicity’s sake will be (somewhat euphemistically) referred to as “optimization experiments” from here forward, can take many forms. For pharmaceuticals, in which optimization is relatively straightforward and commonplace, this could be varying the dose of the drug. There is additional complexity for other interventions, for example, current intensity, frequency, train duration, and location for neuromodulation, or amount, massing, and spacing for behavioral interventions, or things like comorbid conditions that are common to all translational endeavors. To be successful, particularly when outcomes can be paradoxical, defining the conditions in which an approach fails to work is as valuable as defining those that do.

We spent a substantial portion of our research efforts over a number of years, lines of inquiry that continue today, to evaluate the conditions in which VNS does not work (Hays et al. 2023). In reality, these efforts were also intended to identify conditions which were more effective than our standard methodology, but the definition of the conditions that failed turned out to be more useful. These experiments were motivated by neuroscience principles, such as receptor desensitization and the synaptic eligibility trace, but were ultimately pragmatic. We simply varied a parameter over a clinically useful range and examined the impact on the efficacy of VNS (Morrison et al. 2019; Morrison et al. 2020; Buell et al. 2018; Borland et al. 2016).

Defining the conditions over which an approach fails can be exceedingly useful in three ways. First, and most obviously, tracing the contours of the ineffective conditions bounds the effective conditions, crucial knowledge for translation. Defining the range of effective conditions ensures avoidance of ineffective conditions and can also provide a range for varying amongst effective parameters. As an example, if an individual cannot tolerate a particular stimulation frequency, delineation of the range of effective frequencies can facilitate selection of a different parameter likely to be efficacious.

Second, defining failure conditions can give insight into the mechanisms that underlie the therapy. For example, we extensively explored the impact of VNS intensity, which predictably changes the activation of the noradrenergic locus coeruleus, and we characterized an inverted-U relationship between stimulation intensity and VNS efficacy (Hays et al. 2023). These experiments led to predictions about what could mediate such a response, which eventually culminated in identifying an interacting system of noradrenergic receptors that underlie VNS actions (Hays et al. 2023; Tseng et al. 2021). Illustrating the convergence of the utility of these optimization experiments, the identification of this mechanism based on interplay of adrenergic receptors set us on a course to develop a pharmacologically-enhanced VNS paradigm that holds considerable clinical promise.

Third, optimization can identify characteristics that delineate response to therapy. It is well-recognized that the heterogeneity across individuals is a potentially crippling source of variability in clinical trials, and specifying features that define who to include or exclude from a clinical study can be immensely important. In our studies, we sought to evaluate efficacy across injury types, severity, location, chronicity, aging, and task difficulty (Khodaparast et al. 2013; Khodaparast et al. 2014; Khodaparast et al. 2016; Hays et al. 2016; Hays et al. 2014; Meyers et al. 2018; Darrow et al. 2020; Ganzer et al. 2018; Pruitt et al. 2016; Meyers et al. 2019; Darrow et al. 2020; Adcock et al. 2022). The obvious disclaimer to include here is that preclinical models can only take us so far in applying this knowledge to human disorders. Though we certainly agree that context is important for interpretation, we would argue that optimization experiments, by their very nature of internally corroborating across conditions, can be instructive, and our VNS stroke studies in rats have largely been corroborated in clinical analyses (Khodaparast et al. 2013; Khodaparast et al. 2014; Khodaparast et al. 2016; Hays et al. 2016; Meyers et al. 2018; Dawson 2022). Collectively, the (impossibly) large feature space created by the interaction of these conditions cannot plausibly be exhaustively explored, but here careful comparison among projects facilitated by the Easy-Likely-Care ranking process can help to sharpen focus on projects mostly likely to be impactful.

There is an unspoken challenge with optimization experiments, particularly with devices: they generally do not align well with what would be considered fundable. It would be unthinkable to bring a drug to a clinical trial without a dosing study, but this common step of simply identifying the range of conditions where a device would be expected to be effective is generally eschewed. Grant funding, even for programs that are intended to promote translation, often reward novelty and a general sort of “that’s cool” je ne sais quoi, attributes which are either orthogonal to, or at least not aligned with, translation. By contrast, optimization experiments, in large measure, repeat an experiment to establish a known outcome across a range of varying conditions, which are by definition, incremental. We posit that the field, and more importantly, funders, recognize the importance of this effort if we are ever to accelerate progress for medical device development and implementation.

### Forming (and maintaining) a great team

It goes without saying that team science requires a functional team, and a functional team is defined by cohesion amongst its members. Forming collaborations is obviously challenging to broadly define formulaically and will vary by circumstances, personalities, and goals. Below, we round up a few observations that have been important to the formation and maintenance of our team approach, which we hope can provide some fodder for consideration.

The first and most crucial guiding principle we affectionately refer to as a “no jerk” policy (hat tip to Robert Sutton for the original, cruder version (Breakthrough Ideas for 2004 n.d.)). We all have an inherent understanding of the importance of this, though it is not to say that a team cannot be effective with jerks (as an example, Michael Jordan was famously difficult). However, in our experience, long-term scientific success relies heavily on a culture of respect, which is largely dependent on avoiding jerks. A related point is the need to avoid problems that arise from egos. Clear communication of expectations, as well as a general belief in the goodwill and respect of your compatriots, is needed to manage this potential challenge. When a team member other than you is recognized for the groups collective efforts, adopting the perspective that one’s success is everyone’s success is valuable. Conversely, when you are recognized and not other team members, clearly articulating their contributions and expressing gratitude is a good approach. This is not rocket science; your parents taught you this: follow the golden rule.

Collectively, these actions build and maintain trust, another crucial component to a successful team. Trust is necessary both for ensuring a culture in which you can take intellectual risks, but is also practically necessary in the conduct of large complex projects. It simply is not feasible to manage all aspects of a complex project; you must trust your team members to make good decisions. Trust arises organically from respect and good judgement.

Relatedly, though moving away from soft skills, distributed competencies are a core component of team success. A sports analogy is also relevant here: a team of pitchers won’t win a world series. Complementary specialized skills are needed to accomplished complex projects. Distributed competencies can take multiple forms. As a classic example, an effective team may include members with individual, non-overlapping expertise in engineering, medicine, and science. Skills need not be academic or professional to be complementary. For example, a team member skilled with interpersonal communication and another with large organizational management competencies may find synergy. A team isn’t just a group, it’s a group with cohesive skills.

Finally, this cohesion amongst team members requires a unified vision. Using our experience as an example, we have maintained a singular focus on clinical translation. To be clear, unified definitively does not mean homogenized. Our experience has been quite the opposite; instead of serving to push ideas towards a mean, collaboration amongst team members can promote vigorous discussion and generate ideas that are further afield from the mean. Balancing ideation with keeping a focus on a goal is a challenge that requires trust and commitment from all team members, but is also core to serious development and translation.

Finally, and not to sound too flippant, but team science is simply more fun and rewarding. Burnout is a serious problem amongst researchers, and having colleagues to share in both the successes and provide support during the challenges is immensely helpful.
